# The application of enhanced recovery after surgery in total gastrectomy: a propensity score-matched analysis

**DOI:** 10.1186/s12957-023-03034-5

**Published:** 2023-05-16

**Authors:** Kozo Yoshikawa, Mitsuo Shimada, Takuya Tokunaga, Toshihiro Nakao, Masaaki Nishi, Chie Takasu, Hideya Kashihara, Yuma Wada, Toshiaki Yoshimoto

**Affiliations:** The Department of Surgery, The University of Tokushima, 3-18-15, Kuramoto-Cho, Tokushima, 770-8503 Japan

**Keywords:** Gastric cancer, Total gastrectomy, Propensity score matching

## Abstract

**Background:**

This study aimed to investigate the feasibility and safety of our enhanced recovery after surgery protocol including early oral intake and omitting nasogastric tube (NGT) placement after total gastrectomy.

**Methods:**

We analyzed 182 consecutive patients who underwent total gastrectomy. The clinical pathway was changed in 2015, and patients were divided into 2 groups (conventional group and modified group). Postoperative complications, bowel movement, and postoperative hospital stays were compared in the two groups in all cases and propensity score matching (PSM).

**Results:**

Flatus and defecation were significantly earlier in the modified group compared with those in the conventional group (flatus: 2 (1–5) days vs 3 (2–12) days, *p* = 0.03; defecation: 4 (1–14) days vs 6 (2–12) days *p* = 0.04). The postoperative hospital stay was 18 (6–90) days in the conventional group and 14 (7–74) days in the modified group (*p* = 0.009). Days until discharge criteria were met were earlier in the modified group compared with that in the conventional group (10 (7–69) days vs 14 (6–84) days *p* = 0.01). Overall and severe complications occurred in nine patients (12.6%) and three patients (4.2%) in the conventional group and twelve patients (10.8%) and four patients (3.6%) in the modified group, respectively (*p* = 0.70 and *p* = 0.83) in all cases. In PSM, there is no significant difference between the two groups concerning the postoperative complications (overall complication 6 (12.5%) vs 8 (16.7%) *p* = 0.56, severe complications 1 (2%) vs 2 (4.2%) *p* = 0.83).

**Conclusions:**

Modified ERAS for total gastrectomy may be feasible and safe.

## Background

Enhanced recovery after surgery (ERAS) was used in patients with various types of cancer, and it is associated with reduced postoperative morbidity and a shorter hospital stay. The ERAS protocol was also recently implemented in distal gastrectomy and early oral intake contributes to a shorter postoperative hospital stay without increasing complications. Total gastrectomy is more challenging than distal gastrectomy. More extended lymphadenectomy is needed, and the difficulty of esophagojejunal anastomosis is correlated with a higher risk of postoperative complications. There is a few ERAS report with total gastrectomy [1].

A nasogastric tube (NGT) is sometimes routinely used in most gastric cancer patients after total gastrectomy in Japan, and early oral intake could cause anastomotic leakage due to direct stimulation of anastomotic sites and an increase in intraluminal pressure. But it remains unknown whether intraoperative NGT removal and early oral intake after total gastrectomy are feasible and safe [2].

Since 2015, our department has dramatically changed the ERAS protocol after total gastrectomy including omitting NGT placement and allowing early oral intake. We analyzed the feasibility and safety of this clinical protocol in consecutive patients who underwent total gastrectomy and were enrolled in our study. This study aimed to investigate the feasibility and safety of our ERAS protocol including early oral intake and omitting NGT placement after total gastrectomy.

## Methods

### Patients

From January 2013 to December 2021, 182 consecutive gastric cancer patients with total gastrectomy including open and laparoscopic/robotic procedures in the Department of Surgery, Tokushima University, Japan, were enrolled in the present study. Their characteristics were retrospectively reviewed. This study was performed in accordance with the Declaration of Helsinki and approved by the Institutional Review Board of Tokushima University (TOCMS: 3215–1). All patients received a sufficient explanation of the study, and written informed consent was obtained.

### Modified enhanced recovery after surgery protocol

A modified ERAS protocol was used for total gastrectomy patients. The clinical procedure was modified in October 2015, and the details of the modified ERAS are shown in Table 1. The main points that were changed are as follows: the NGT was removed during surgery, and on the other hand, NGT was removed postoperative day (POD) 1 in the conventional group; postoperative fluid intake was performed on POD 1; and herbal medicine (Dai-kenchu-to) was administered to all patients.

**Table 1:** Clinical pathway details

	Conventional group	Modified group
Perioperative		
Counseling	Pulmonary rehabilitation	Pathway explanation and informative booklet
Intraoperative		
Analgesia	Not standardized	Multimodal: TAP block for laparoscopic surgery + CNS-targeted drugs
Prophylaxis	Antibiotic prophylaxis, VTE (pharmacological and mechanical)	Antibiotic prophylaxis, VTE (pharmacological and mechanical)
Fluids	Not standardized	Goal-directed fluid management
Extubation	Immediate extubation	Immediate extubation
NGT	Remove on POD 1	Remove at the end of surgery
Postoperative		
Analgesia	Not standardized	NSAIDs
Fluid	Not standardized	Zero balance goal; stop iv fluid within POD 4
Abdominal drain	Always placed	Always placed. No routine anastomotic leak test. Removed on POD 5
Line management	Not standardized	Remove urinary catheter on POD 2
Diet	POD 2 clear fluids, POD 4 soft diet	POD 1 clear fluids, POD 2,3 nutritional supplement, POD 4 soft diet
Rehabilitation	Not standardized	POD 1–3 pulmonary physiotherapy POD 1 chair and bedside exercise POD 2–3 assisted ambulation
Herbal medicine	None	Dai-kenchu-to (15 g/day)

*TAP* transversus abdominis plane, *CNS* central nervous system, *VTE* venous thromboembolism, *NGT* nasogastric tube, *POD* postoperative day

Patients were usually admitted 1 day before surgery, and they could eat a regular diet until lunchtime. Bowel preparation was performed including magcorol 1 pack and sennoside 2 T in both groups.

General anesthesia was performed using a transversus abdominis plane block in the modified procedure group. The NGT was removed during the surgery in the modified group. Patients were allowed to drink water on POD 1 and take a nutritional supplement on POD 2 and POD 3. They could begin eating soft foods on POD 4, with a more solid diet served each subsequent day. Drain amylase levels were measured on POD 1 and 3. Blood tests were performed on POD 1, 3, and 5 or 7. The drainage tube was removed on POD 4. Patients received nutritional education before they were discharged. Patients were discharged after they met the following discharge criteria: normal laboratory test results, normal body temperature, controlled pain, adequate mobility, and sufficient oral food intake. However, patients’ discharge was influenced by the patient’s requests and the hospital’s policies. The ERAS protocol was used for 182 consecutive patients who underwent total gastrectomy with no exclusion criteria.

### Dai-kenchu-to

Dai-kenchu-to (DKT) is a traditional Japanese medicine originally described in a Chinese classic article and independently developed in Japan. It is a mixture of extract powders from dried Japanese pepper, processed ginger, ginseng radix, and malt sugar powder and is reported to have the effects of improving gastrointestinal motility, activating anti-inflammatory, increasing intestinal blood flow, and altering the intestinal microbiome [3, 4].

### Definition of complications

Postoperative complications were defined in accordance with the Clavien–Dindo classification system (grade IIIA or higher complications were considered to be severe complications) [5]. Death from any cause within the postoperative 30 days was defined as hospital mortality. Hospital admission for any cause within 30 days after discharge was defined as readmission.

### Stage and surgical plan

All patients underwent radical total gastrectomy in accordance with the treatment guidelines of the Japanese Gastric Cancer Association (JGCA) [6]. The clinicopathological TNM stage and the tumor regression grade were evaluated in accordance with the JGCA classification of gastric carcinoma [7].

The standard surgical strategy for advanced gastric cancer was open total gastrectomy with D2 lymphadenectomy. For the patient who had a tumor with a greater curvature or when there was suspicion of lymph node metastasis, a splenectomy was performed. A laparoscopic or robotic approach was applied for cStage I cases. For patients with cStages II–IV, the patient’s case was discussed at the department’s council meeting, and a decision was made on the preferred surgical approach on a case-by-case basis.

### Operative procedure

Roux-en-Y reconstruction methods were used in all surgical procedures. For esophagojejunal anastomosis, a circular stapler was used in the open gastrectomy and a linear or circular stapler was used for laparoscopic/robotic approaches [8, 9]. A drain was placed behind the esophagojejunal anastomosis. Using the laparoscopic or robotic approach, Japan Society of Endoscopic Surgery-qualified surgeons performed the total gastrectomy as a surgeon or as a first assistant.

### Statistical analyses

Data were analyzed using the JMP statistical software program (SAS Institute Inc., Cary, NC, USA). The *χ*^2^ test or Fisher’s exact test was used to compare the categorical variables. The Mann–Whitney *U* test was used to compare the continuous variables. Quantitative variables are presented as the mean ± standard deviation. A *p* value of < 0.05 was considered statistically significant.

### Propensity score matching

Propensity score matching (PSM) analysis was used with the following factors: age, sex, body mass index, pathological stage, surgical approach, combined surgery, and lymph node dissection. These factors were selected by univariate analysis. We performed 1:1 matching using a 0.20-caliper width.

## Results

### Patient characteristics

The patients’ characteristics are shown in Table 2. In the conventional group, the more advanced cases were included. In the modified group, minimal invasive surgery (MIS) included patients who underwent laparoscopic or robotic surgery. The extent of lymph node dissection was not significantly different between the two groups. Surgical time and intraoperative blood loss were decreased in the modified group compared (321 ± 9.4 min, 73 ± 19 ml) with those in the conventional group (347 ± 9.8 min 229 ± 20 ml). Open total gastrectomy was performed in 44 patients, while laparoscopic total gastrectomy was performed in 27 patients in the conventional group. Open total gastrectomy was performed in 28 patients, while laparoscopic total gastrectomy was performed in 45 patients, and robotic total gastrectomy was performed in 38 patients in the modified group. The extent of lymph node dissection was D1 + in 26 patients, D2 in 44 patients, and D2 + in one patient in the conventional protocol group and D1 + in 35 patients, D2 in 75 patients, and D2 + in one patient in the modified group.

**Table 2:** Characteristics of all patients

		Conventional group (*n* = 71)	Modified group (*n* = 111)	*p* value
Age		67.4 ± 1.36	67.3 ± 1.08	0.93
Sex	Male	57	79	0.17
	Female	14	32	
BMI		23.5 ± 0.4	22.6 ± 0.4	0.08
Pathological stage	0	1	8	< 0.01
	I	22	37	
	II	22	29	
	III	13	35	
	IV	13	2	
Approach	Open	44	28	< 0.01
	Lap	27	45	
	Robotic	0	38	
LN dissection	D1 +	26	35	0.72
	D2	44	75	
	D2 +	1	1	
Combined surgery		13	5	< 0.01
	GB	4	2	
	Spleen	7	3	
	Liver	1	0	
Operation time (min)		347 ± 9.8	321 ± 9.4	0.03
Intraope blood loss (ml)		229 ± 20	73 ± 19	< 0.01

*BMI* body mass index, *Lap* laparoscopic, *LN* lymph node, *Intraope* intraoperative, *GB* gallbladder

### Postoperative results

Because the strategy was changed for the modified clinical procedure, patients with ERAS were able to drink water earlier than those in the modified group, while flatus and defecation were noted significantly earlier in the modified group compared with those in the conventional group (flatus: 2 (1–5) days vs 3 (2–12) days, *p* = 0.03; defecation: 4 (1–14) days vs 6 (2–12) days, *p* = 0.04). The postoperative hospital stay was 18 (6–90) days in the conventional group and 14 (7–74) days in the modified group (*p* = 0.009). Next, we check the day when they met the discharge criteria, as described above, and we reviewed the discharge met day criteria retrospectively. The discharge met day was earlier in the modified group compared with that in the conventional group (10 (7–69) days vs 14 (6–84) days, *p* = 0.01). Overall complications and severe complications occurred in 9 patients (12.8%) and 3 patients (4.2%) in the conventional group and 12 patients (10.8%) and 4 patients (3.6%) in the modified group, respectively. One patient died due to aortic dissection, and the clinical pathway protocol was completed by 59 patients (84.2%) and 97 patients (87.3%), respectively. There was no significant difference in readmission or NGT reinsertion between the two groups (Table 3).

**Table 3:** Perioperative results in all patients

	Conventional group (*n* = 71)	Modified group (*n* = 111)	*p* value
Initial day of flatus (day)	3 (2–12)	2 (1–5)	0.03
Initial day of defecation (day)	6 (2–12)	4 (1–14)	0.04
Postope hospital stays (day)	18 (6–90)	14 (7–74)	< 0.01
Fulfill discharge criteria (day)	14 (6–84)	10 (7–69)	0.01
Complication (CD > 2)	9	12	0.70
Abdominal abscess	2	0	
Postoperative bleeding	0	1	
Stenosis	2	2	
Ileus	0	4	
Esophagojejunal leakage	2	0	
Pneumonia	2	0	
Duodenal leakage	0	1	
Pancreatic fistula	1	4	
Severe complications (CD > 3)	3	4	0.83
Reinsertion of NG tube	2	2	0.65
Mortality	1	0	
Readmission	2	0	0.05
Completion of clinical pathway	59	97	0.42

*Postope* postoperative

### Propensity score matching

To further determine the usefulness of modified ERAS, we used PSM to balance the differences between the two groups. After screening and matching, 96 patients were included, and 48 patients were assigned to each group. The patients’ characteristics after PSM are shown in Table 4. Initial flatus was significantly earlier, and defecation tended to be earlier in the ERAS group compared with those in the conventional group (flatus: 2 (1–5) days vs 3 (2–11) days, *p* = 0.04; defecation: 4 (1–14) days vs 5 (2–12) days, *p* = 0.11). Overall and severe complications occurred in six patients (12.5%) and one patient (2%) in the conventional group and eight patients (16.6%) and two patients (4.2%) in the modified group, respectively (*p* = 0.56 and *p* = 0.83). Clinical protocol completion was achieved in 40 (83.3%) patients and 39 (81.2%) patients in the conventional and modified groups, respectively. There was no significant difference between the two groups for readmission or NGT reinsertion (Table 5).

**Table 4:** Characteristics of patients with PSM

		Conventional group (*n* = 48)	Modified group (*n* = 48)	*p* value
Age		65.9 ± 1.66	67.9 ± 1.64	0.38
Sex	Male	35	35	0.95
	Female	13	13	
BMI		23.7 ± 0.51	23.4 ± 0.65	0.77
Pathological stage	0	1	1	1.00
	I	18	18	
	II	15	15	
	III	12	12	
	IV	2	2	
Approach	Open	23	22	0.83
	Lap/Robo	25	26	
LN dissection	D1 +	18	14	0.36
	D2	30	33	
	D2 +	0	1	
Combined surgery		1	2	0.55
	GB	0	0	
	Spleen	1	2	
	Liver	0	0	
Operation time (min)		339 ± 9.8	310 ± 12	0.07
Intraope blood loss (ml)		177 ± 25	97 ± 31	0.05

*BMI* body mass index, *Lap* laparoscopic, *Robo* robotic, *LN* lymph node, *Intraope* intraoperative, *GB* gallbladder

**Table 5:** Perioperative results in patients with PSM

	Conventional group (*n* = 48)	Modified group (*n* = 48)	*p* value
Initial day of flatus (days)	3 (2–11)	2 (1–5)	0.04
Initial day of defecation (days)	5 (2–12)	4 (1–14)	0.11
Postope hospital stays (days)	17 (6–63)	16 (7–74)	0.62
Fulfill discharge criteria (days)	12 (6–58)	12 (7–69)	0.77
Complication (CD > 2)	6	8	0.56
Abdominal abscess	2	0	
Postoperative bleeding	0	1	
Stenosis	2	0	
Ileus	0	4	
Esophagojejunal leakage	0	0	
Pneumonia	1	0	
Duodenal leakage	0	0	
Pancreatic fistula	1	3	
Severe complications (CD > 3)	1	2	0.83
Reinsertion of NG tube	1	1	1.00
Mortality	1	0	
Readmission	2	0	0.09
Completion of clinical pathway	40	39	0.60

*Postope* postoperative

## Discussion

In the present study, the NGT was removed from all patients in the modified group at the end of the surgery, and oral intake was started on POD 1. The modified ERAS protocol including omitting the NGT and early oral intake did not increase postoperative complications even in the PSM analysis. In all patient analyses, the postoperative time when the discharge criteria were met was shorter in the modified group compared with that in the conventional group.

In general, the length of postoperative hospital stays and reduction in hospital costs were the main outcomes of ERAS. Our hospital is in a rural city, and older patients were enrolled in this study. Thus, the patients tended to remain in the hospital after meeting the discharge criteria. It is difficult for patients to return home under the clinical protocol procedures. Although the duration of the postoperative hospital stay was shorter in the modified group compared with that in the conventional group, the hospital stay was longer than that in high-volume centers in Japan [10, 11]. Long hospital stays are also attributed to the Japanese Diagnosis Procedure Combination-based Payment System. Thus, we investigated the day at which the discharge criteria were met. Although there was no statistically significant difference in PSM, the time until the discharge criteria were met was shorter in the modified group compared with that in the conventional group in all patient analyses. We believe that the improvement in patient care obtained by implementing ERAS contributed to reducing the length of the hospital stay.

Early postoperative oral feeding accelerates the patient’s recovery after gastrectomy [12]. However, it is difficult to implement oral feeding after total gastrectomy because of concerns that early food intake would allow the passage of food near the anastomotic site and that the intraluminal pressure would increase, which could result in anastomotic leakage. Many surgeons routinely restrict oral intake in patients receiving conventional care. Recently, patients in Japan have generally been allowed to start oral feeding on an early POD [1]. Total gastrectomy is likely to be more complicated than distal gastrectomy because it is difficult to perform esophagojejunostomy; however, there is no definite evidence to support this hypothesis for total gastrectomy. Patients start oral food intake at POD 2 as part of our conventional management, but after ERAS protocol modification, patients started the oral food intake on POD 1. There was no anastomotic leakage in the modified group. ERAS, unlike conventional care, does not require long postoperative fasting periods. A recent prospective study confirmed that early oral food intake after laparoscopic gastric surgery is safe and might be associated with enhanced recovery with a shorter hospital stay [13–15]. The ERAS guidelines for gastrectomy also recommend early initiating postoperative oral nutrition, but a delay in bowel recovery may hamper early oral nutrition in some patients after gastrectomy. However, there are no serious compilations associated with early oral nutrition occurred, such as aspiration or anastomosis leakage. Therefore, if careful monitoring is performed, ileus may not be a major concern when implementing early oral nutrition in patients who have undergone total gastrectomy.

Many surgeons think that NGT is useful to reduce the passage of food by the anastomotic site and to maintain calm conditions near the anastomotic site. But, in bariatric surgery, routine placement of NGT in patients operated on laparoscopic sleeve gastrectomy was not useful in reducing anastomotic leakage [16]. It is unclear whether early removal of NGT prevents anastomotic leakage and accelerates the recovery of bowel movement. This study showed that the following gastrectomy, omitting NGT placement after surgery was safe, and there was no increase in anastomotic-related complications. In this study in the modified group, four patients experienced postoperative ileus, and two patients required NGT reinsertion, but these patients recovered without surgery. Prolonged perioperative fasting and NGT are likely to induce nausea and delay bowel function recovery, and the patients without a postoperative NGT recovered postoperative bowel movement earlier than patients with an NGT and that routine postoperative NGT intubation is unnecessary after elective surgery [17]. Although previous reports suggested omitting NGT placement does not enhance the postoperative recovery in gastric cancer [17], our modified ERAS included that omitting NGT enhanced the postoperative recovery. It is speculated that this is the effect of multiple items such as early oral intake, reduction of intestinal edema due to early termination of infusion, and administration of herbal medicine: Dai-kenchu-to.

The first flatus and defecation indicate recovery of bowel movement, and these factors occurred earlier in the modified group than in the conventional group in our study. A previous report suggested that the first day of flatus after gastric surgery was earlier in patients who underwent modified ERAS care than in those who received conventional care [18]. After modifying the ERAS protocol, many additional modifications were made including administering herbal medicine, Dai-kenchu-to medicine, routinely to reduce the time to first flatus and defecation. DKT has been clinically used to treat various gastrointestinal diseases, including postoperative ileus, abdominal bloating, and cold sensation in the abdomen. DKT has specific functions such as improving intestinal movement, increasing colonic blood flow, and suppressing inflammation. These effects may influence the early recovery from the postoperative course [3, 4].

ERAS factors include early ambulation, early postoperative nutrition, and early elements that can contribute together to reducing the length of the hospital stay without increasing postoperative complications [19]. Other factors such as MIS may also have influenced the length of stay [20, 21]. Thus, we performed PSM to eliminate the effects of the patient’s background. In the ERAS group, patients with more advanced cancer were treated, and minimally invasive surgery including laparoscopic and robotic surgery was performed frequently. However, even after PSM, there was no increase in the number of postoperative complications, although there was no significant shorter hospital stay.

Recently, there have been many older patients who underwent total gastrectomy, and an early recovery prevents postoperative complications from developing in these patients. Thus, more ERAS should be performed, and the postoperative activity must be increased.

In Asian countries, ERAS was analyzed frequently and had a positive effect on the postoperative course [19]. On the other hand, in European countries, ERAS was not prevalent [22]. But recently, Spanish institutions reported the usefulness of ERAS [23]. Although gastric cancer is more common in Asian countries, this ERAS will be more popular in European countries.

This study had several limitations. The sample size was small, the study had a retrospective design, and there was no randomization for the two treatment arms.

## Conclusions

The findings of this study revealed that modified ERAS including early oral intake and omitting NGT placement for total gastrectomy may be feasible and safe.

## Data Availability

The datasets used during the current study are available from the corresponding author upon reasonable request.

## Abbreviations

ERAS: Enhanced recovery after surgery
NGT: Nasogastric tube
MIS: Minimally invasive surgery
POD: Postoperative day
